# Excellent Outcomes and High Rates of Return to Duty but a 10% Chance of Tear Progression Are Seen Following Arthroscopic Debridement Alongside Adjunctive Procedures for Articular‐Sided, Partial‐Thickness Supraspinatus Tears (Ellman Grade I) in Active‐Duty Service Members Younger Than 50 Years

**DOI:** 10.1002/ars2.70011

**Published:** 2026-06-10

**Authors:** Brian Skura, Ian S. Rice, Alexis B. Sandler, Samuel Howard, Richy Charls, John Tyler, John P. Scanaliato, Ron Gilat, Nata Parnes

**Affiliations:** ^1^ Beacon Orthopaedics and Sports Medicine Cincinnati Ohio U.S.A.; ^2^ William Beaumont Army Medical Center El Paso Texas U.S.A.; ^3^ Maimonides Health Brooklyn New York U.S.A.; ^4^ Carthage Area Hospital Carthage New York U.S.A.

## Abstract

**Purpose:**

To evaluate clinical outcomes and return‐to‐duty rates in active‐duty service members younger than 50 years after arthroscopic debridement of Ellman grade I, articular‐sided partial‐thickness rotator cuff tears (PT‐RCTs) of the supraspinatus.

**Methods:**

A retrospective review of active‐duty service members under 50 who underwent arthroscopic debridement of Ellman grade I PT‐RCTs without associated cartilage lesions, labral repair, or rotator cuff repair between January 2014 and June 2022 with ≥24 months of follow‐up was conducted. Outcomes included pain Visual Analog Scale (VAS), Single Assessment Numeric Evaluation (SANE), and American Shoulder and Elbow Surgeons (ASES) scores; achievement of minimal clinically important difference (MCID) (½ standard deviation method); and range of motion (ROM), return to activity, adverse events, and tear progression.

**Results:**

In total, 67 patients met inclusion criteria (mean age 39.6 ± 7.1 years, 59.7% male, 5.6 ± 2.4 years of follow‐up). All patients underwent adjunctive subacromial decompression, 56.7% biceps tenotomy/tenodesis, 37.3% acromioclavicular joint resection, and 17.9% rotator interval release. Significant improvements in pain and functional outcomes were observed postoperatively (VAS: 8.1 ± 1.4 to 1.7± 2.5, *P* < .0001; SANE: 49.0 ± 19.2 to 84.7 ± 16.5, *P* < .001; ASES: 47.6 ± 10.23 to 87.8 ± 14.6, *P* < .001), and the MCID was achieved in most patients (VAS: 92.5%, SANE 82.1%, ASES: 89.6%). ROM did not change significantly. Return to unrestricted active duty was achieved by 88% of patients (n = 59) and preinjury level of sport by 85.1% (n = 57). S patients (10.5%) progressed to symptomatic full‐thickness rotator cuff tears that warranted rotator cuff repair.

**Conclusions:**

Arthroscopic debridement of Ellman grade I, articular‐sided supraspinatus PT‐RCT with adjunctive procedures in active‐duty service members younger than 50 years shows substantial pain relief, functional recovery, and return to military service and sport. Despite improvements in pain relief and functional outcomes, approximately 1 in 10 patients experienced atraumatic tear progression to a symptomatic full‐thickness rotator cuff tear and ultimately underwent rotator cuff repair.

**Level of Evidence:**

Level IV, retrospective therapeutic case series.

## Graphical abstract

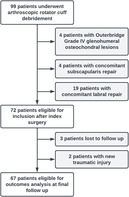

Rotator cuff pathology is a significant source of shoulder pain and dysfunction in active‐duty military personnel.^1^, ^2^, ^3^, ^4^ Ellman grade 1 partial‐thickness rotator cuff tears (PT‐RCT), defined as tears less than 3 mm (<25% of tendon thickness), represent a mild but clinically relevant injury on the rotator cuff spectrum of pathology.^5^

Conservative management is classically the first line of treatment for low‐grade PT‐RCT;^6^, ^7^ however, the role of arthroscopic debridement is emerging as a viable treatment option for this pathology, especially among younger, physically active populations.^8^, ^9^, ^10^, ^11^, ^12^ In their comprehensive reviews on the arthroscopic management of isolated PT‐RCT, Longo et al. and Strauss et al. found debridement alone to be an appropriate treatment option for Ellman grades I‐II PT‐RCTs in terms of pain and function, although tear progression remains a possibility.^10^, ^13^ Transtendinous, transosseous, or rotator cuff takedown and repair remain an option for PT‐RCT; however, repair is typically reserved for tears of > 50% of the tendon thickness rather than tears involving <25% of the tendon.^13^, ^14^

Given the unique occupational requirements and increased shoulder demand characteristic of active‐duty service members (ADSM), further research is needed to establish the most effective treatment protocols for low‐grade, partial‐thickness supraspinatus tears in this population. Additionally, rates for tear progression as well as return to duty in this population remain unclear. The purpose of this study was to evaluate clinical outcomes and return‐to‐duty rates in ADSM younger than 50 years after arthroscopic debridement of Ellman grade I, articular‐sided PT‐RCT involving the supraspinatus. The authors hypothesized that debridement of this specific PT‐RCT pattern will offer improved pain relief and PROMs as compared with preoperatively.

## METHODS

### Inclusion and Exclusion Criteria

This study was approved by the Institutional Review Board (IRB 2024‐0006). This study is a retrospective review of active‐duty military personnel aged 18 to 49 years who underwent arthroscopic debridement of Ellman grade I, articular‐sided supraspinatus PT‐RCT between January 2014 and June 2022 with at least 24 months of follow‐up. All patients underwent a minimum 3‐month trial of nonoperative management with guided physical therapy prior to proceeding with surgery. Patients with glenohumeral osteochondral defects were excluded given the association with cartilage defects and decreased rates of return to duty after rotator cuff repair.^15^ Additionally, patients presenting with a full‐thickness rotator cuff tear who subsequently underwent repair or those who underwent labral repair were excluded to standardize patients to those who had not required extensive immobilization postoperatively, with extensive immobilization defined as 6 weeks of mandatory immobilization (as compared with 2 weeks of mandatory immobilization after biceps tenodesis per the senior surgeons’ preferred postoperative protocol). Patients who sustained new, ipsilateral traumatic shoulder injuries postoperatively were reported but excluded from outcomes analysis to best estimate the risk of atraumatic tear progression.

### Surgical Indications

Prior to surgery, all patients who presented with shoulder pain underwent a minimum 3‐month trial of nonoperative management with guided physical therapy, anti‐inflammatory medication use, and activity modification. Patients were ultimately indicated for surgery if they failed the 3‐month trial of nonoperative management and endorsed continued pain and activity or occupational limitations unrelieved or unacceptably relieved by nonoperative treatment. There were no corticosteroid injections administered during the trial of nonoperative management. Although surgical indications varied, all patients who were indicated for shoulder diagnostic arthroscopy and intraoperatively were found to have an Ellman grade I, articular‐sided PT‐RCT that was subsequently debrided were included.

In total, 99 patients underwent arthroscopic debridement of Ellman grade I articular‐sided PT‐RCT; however, 4 had concomitant glenohumeral cartilage lesions, 4 underwent concomitant subscapularis repair, 19 underwent concomitant labral repair, and 3 were lost to follow‐up. Postoperatively, 2 patients sustained new traumatic injuries to their operative shoulder and were excluded from analysis: 1 from an improvised explosive device blast and another from a fall from a height sustained during a military training exercise. Ultimately, 67 patients were eligible for inclusion.

### Surgical Technique

All surgical procedures were performed by the senior shoulder and elbow fellowship‐trained surgeon (N.P.). All patients underwent an interscalene block and were positioned in the beach chair position. Following diagnostic arthroscopy, the supraspinatus tendon tear was visualized, measured with an arthroscopic probe with 1‐mm graduated scale markings, and debrided to the level of healthy tissue using an arthroscopic shaver. Concomitant procedures were performed as indicated by intraoperative findings. Biceps tenodesis was indicated for all patients with SLAP lesions, intraoperative inflammation or partial tearing of the long head of the biceps tendon, and/or instability of the biceps tendon. Acromioclavicular joint resection (ACJR) was indicated for all patients with radiographic evidence of acromioclavicular (AC) joint arthritis as well as clinical evidence of ACJ pain including (1) point tenderness along the AC joint that was not present on the contralateral side, (2) positive cross body adduction test, and/or (3) positive high AC joint test. Arthroscopic rotator interval release (ARIR) was indicated for any patients who showed diminished passive external rotation with or without diminished passive forward flexion as compared with the contralateral side and/or visualized scar tissue or adhesions within the rotator interval intraoperatively. All patients showed subacromial bursitis and subsequently underwent arthroscopic subacromial decompression (ASAD). After ASAD was complete, the tear was marked from the articular side and evaluated from the subacromial view and the bursal aspect of the tendon was confirmed to be intact and stable to probing to ensure that the tear only involved the articular surface of the tendon.

Postoperative rehabilitation protocols included active and active‐assisted full range of motion (ROM) for 6 weeks beginning immediately after the interscalene block subsided. By 6 weeks, patients progressed to a gradual strengthening protocol.

### Data Collection

Data including patient‐reported outcome measures (PROMs), ROM, military occupational specialty, return to activity, surgical complications, and progression to full‐thickness tendon tear were collected and analyzed. Pain was measured using the Visual Analog Scale (VAS). Function was measured using the American Shoulder and Elbow Surgeons (ASES) score and the Single Assessment Numeric Evaluation (SANE) score. Active ROM in forward flexion (FF), external rotation (ER), and internal rotation (IR) were collected by the operating surgeon or the clinic's physician assistant, with FF and ER measured using a goniometer and IR graded by the highest vertebral level the patient could reach with the thumb. ROM data were collected preoperatively as well as at the unstandardized latest postoperative follow‐up appointment for each patient. Return to activity included both return to unrestricted military duty and return to sport activity, with return to preinjury activities defined as the ability to participate at the same level of intensity and function as before the injury. Return to preinjury activity data were collected as binary variables. Progression of partial‐ to full‐thickness rotator cuff tears was evaluated by follow‐up imaging when clinically indicated due to persistent pain and rotator cuff symptomology as well as supraspinatus weakness (4/5 strength or less). Patients who were clinically suspected to have symptomatic progression to full‐thickness rotator cuff tears underwent magnetic resonance imaging to confirm the diagnosis and subsequently were indicated for arthroscopic rotator cuff repair.

### Statistical Analysis

Descriptive statistics were used to summarize demographic and clinical characteristics. Continuous variables were presented as mean ± standard deviation, and categorical variables were presented as frequencies and percentages. The minimal clinically important difference (MCID) was calculated using the one‐half standard deviation technique, and rates of achieving this threshold of change were presented as percentages. Paired t‐tests were used to compare preoperative and postoperative outcomes, including VAS, SANE, ASES, ROM (FF, ER, IR), and *P* values were calculated to assess statistical significance. Statistical significance was defined as a *P* value of less than 0.05. All statistical analyses were performed using SPSS software version 25.0 (IBM, Armonk, NY, USA).

## RESULTS

In total, 99 patients underwent arthroscopic debridement of Ellman grade I articular‐sided PT‐RCT; however, 4 had concomitant glenohumeral cartilage lesions, 4 underwent concomitant subscapularis repair, 19 underwent concomitant labral repair, and 3 were lost to follow‐up. Postoperatively, 2 patients sustained new traumatic injuries to their operative shoulder and were excluded from analysis: 1 from an improvised explosive device blast and another from a fall from a height sustained during a military training exercise.

**FIGURE 1:**
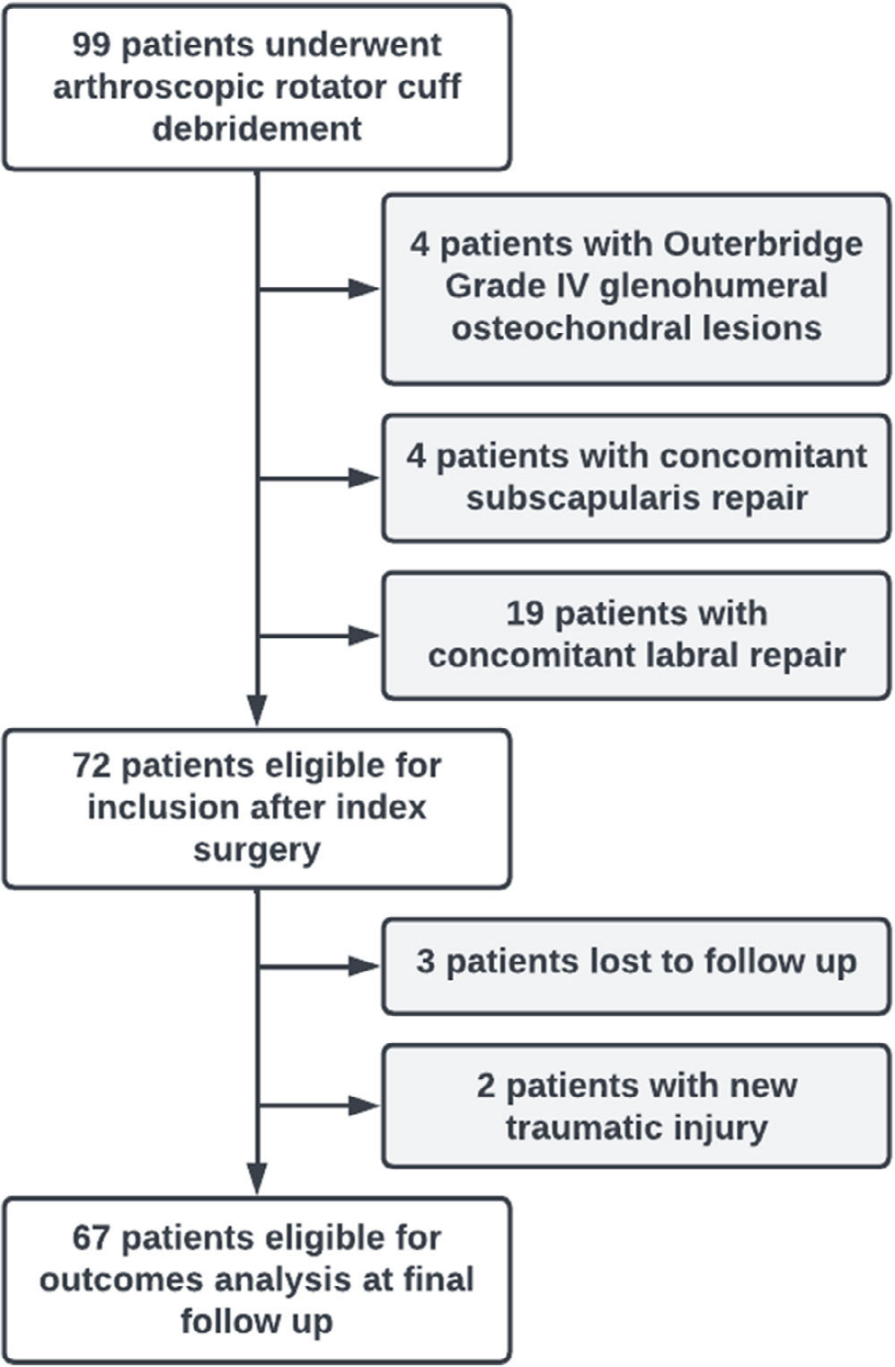
Patient inclusion flowchart.

In total, 67 patients were eligible for inclusion in final analysis (Figure 1). Patient demographics are presented in Table 1 and occupational specialties in Table 2. The mean duration of symptoms was 30.9 ± 53.2 months (range: 3‐348), and the mean follow‐up period was 5.6 ± 2.4 years (range: 2‐10 years). The majority of procedures involved the right shoulder (n = 39, 58.2%) and the dominant extremity (n = 42 patients, 62.7%). The mean tear size was 1.6 ± 0.63 mm. All 67 patients underwent ASAD in addition to supraspinatus tendon debridement. Other concomitant procedures included biceps tenotomy or tenodesis in 60 patients (89.6%), ACJR in 25 patients (37.3%), and ARIR in 15 patients (22.4%), and specific combinations are presented in Table 1. The most common combination of adjunctive procedures included ASAD combined with biceps tenotomy or tenodesis at 41.8% (n = 28) followed by ASAD, biceps tenotomy or tenodesis, and ACJR at 28.4% (n = 19).

**TABLE 1:** Study Population Characteristics: 67 Patients

	Value
Total patients, n	67
Demographics	
Age, years	39.61 ± 7.14 (range: 21‐49)
Male, n (%)	40 (59.7%)
Right‐sided pathology, n (%)	39 (58.2%)
Dominant arm, n (%)	42 (62.7%)
Traumatic mechanism, n (%)	25 (37.3%)
Time to surgery, months	30.9 ± 53.2 (range: 3‐348)
Mean tear size, mm	1.6 ± 0.63 mm
Follow‐up, months	67.7 ± 28.5 (range: 24‐120)
Concomitant procedures: general	
Arthroscopic subacromial decompression (ASAD), n (%)	67 (100%)
Biceps tenotomy/Tenodesis (BT), n (%)	60 (89.6%)
Acromioclavicular joint resection (ACJR), n (%)	25 (37.3%)
Arthroscopic rotator interval release (ARIR), n (%)	15 (22.4%)
Concomitant procedures: specifics	
ASAD, n (%)	3 (4.5%)
ASAD + BT, n (%)	28 (41.8%)
ASAD + ACJR, n (%)	2 (3.0%)
ASAD + ARIR, n (%)	1 (1.5%)
ASAD + BT + ACJR, n (%)	19 (28.4%)
ASAD + BT + ARIR, n (%)	10 (14.9%)
ASAD + ACJR + ARIR, n (%)	1 (1.5%)
ASAD + BT + ACJR + ARIR, n (%)	3 (4.5%)

**TABLE 2:** Study Population Military Occupational Specialties

**Military Occupational Specialty**	**N (%)**
Infantry	36 (53.7%)
Military police	6 (8.9%)
Artillery	5 (7.5%)
Firefighter	5 (7.5%)
Intelligence	4 (6.0%)
Driver	3 (4.5%)
Mechanic	3 (4.5%)
Engineer	2 (3.0%)
Food services	2 (3.0%)
Linguist	1 (1.5%)

### Patient‐Reported Outcome Measures (PROMs)

Significant improvements were observed in pain and shoulder function postoperatively (Table 3). The mean VAS score improved from 8.1 ± 1.4 preoperatively to 1.7 ± 2.5 postoperatively (*P* < .001). Similarly, the mean SANE score increased from 49.0 ± 19.2 preoperatively to 84.7 ± 16.5 postoperatively (*P* < .001), and the ASES score improved from 47.6 ± 10.3 to 87.8 ± 14.6 (*P* < .001).

**TABLE 3:** Patient‐Reported Outcome Measures (PROMS) and Range of Motion (ROM)

	Preoperative	Postoperative	*P* Value
PROMs			
VAS	8.1 ± 1.4	1.7 ± 2.5	<.001*
SANE	49.0 ± 19.2	84.7 ± 16.5	<.001*
ASES	47.6 ± 10.3	87.8 ± 14.6	<.001*
ROM			
FF, °	157.0 ± 5.2	157.7 ± 5.8	.405
ER, °	68.3 ± 4.3	67.3 ± 8.7	.381
IR, T‐level	T9.0 ± 2.5	T8.6 ± 2.3	.265

ASES, American Shoulder and Elbow Surgeons score; ER, external rotation; FF, forward flexion; IR, internal rotation; SANE, Single Assessment Numeric Evaluation; VAS, Visual Analog Scale.

* Indicates statistical significance.

For VAS, the MCID was calculated to be 1.5 and 92.5% (n = 62) of patients achieved this level of improvement. For SANE, the MCID was calculated to be 12.5 and 82.1% (n = 55) of patients achieved this level of improvement. For ASES, the MCID was calculated to be 13.4 and 89.6% (n = 60) of patients achieved this level of improvement.

Disaggregated data by sex are presented in Table 4. SANE scores were noted to be significantly lower among male than among female patients, whereas VAS and ASES scores did not show statistically significant differences.

**TABLE 4:** Disaggregated Data by Sex

	Male (N = 42)	Female (N = 25)	*P* Value
Postoperative patient‐reported outcome measures†			
VAS	1.7 ± 2.5	0.72 ± 1.4	.078
SANE	83.5 ± 16.9	92.0 ± 9.2	.024*
ASES	88.6 ± 13.0	93.4 ± 8.4	.114
Postoperative range of motion†			
FF, °	157.6 ± 3.9	157.7 ± 8.2	.939
ER, °	66.9 ± 6.2	67.1 ± 12.3	.922
IR, T‐level	T9.1 ± 2.2	T8.1 ± 2.3	.118
Rates of progression to symptomatic tear progression indicated for repair	7 (16.7%)	0 (0%)	.040*

ASES, American Shoulder and Elbow Surgeons score; ER, external rotation; FF, forward flexion; IR, internal rotation; SANE, Single Assessment Numeric Evaluation; VAS, Visual Analog Scale.

* Indicates statistical significance.

† Indicates calculations performed with patients who did not progress to failure (N = 35 for male patients, N = 25 for female patients).

### ROM

There were no statistically significant changes in ROM observed postoperatively (Tables 3 and 4).

### Return to Activity

At final follow‐up, 88.1% (n = 59/67) of patients returned to unrestricted active duty. Similarly, 85.1% (n = 57/67) of patients were able to return to their preinjury level of sport/recreational activities.

### Adverse Events and Tear Progression

**FIGURE 2:**
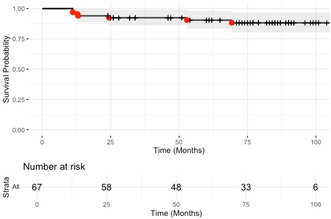
Survival analysis for patients who progressed to full‐thickness rotator cuff tear and underwent rotator cuff repair. Black tick marks indicate final follow‐up for patients who did not progress to rotator cuff repair; red circles indicate time points at which time patients who progressed to symptomatic full‐thickness tears underwent rotator cuff repair.

There were no cases of postoperative infection or nerve injury observed among the patient cohort. Ultimately, 7 patients (10.4%) experienced atraumatic, symptomatic tear progression to a full‐thickness rotator cuff tear by the final follow‐up period and were indicated for rotator cuff repair. Patients who progressed to full‐thickness rotator cuff tear were significantly younger with a greater male and left‐sided predominance as compared with patients who did not progress, and a statistically significant difference was observed between male and female patients (Tables 4 and 5). A survival curve for the analysis is presented in Figure 2. Although preoperative PROM values were similar between the cohorts, postoperative VAS, SANE, and ASES showed significantly greater improvements in patients that did not progress to full‐thickness rotator cuff tear. Other than symptomatic tear progression, there were no additional reoperations indicated among the patient cohort.

**TABLE 5:** Comparative Analysis

Total Patients, n	Patients With Symptomatic Tear Progression Indicated for Repair (n = 7)	Patients Without Symptomatic Tear Progression (n = 60)	*P* Value
Demographics			
Age, years	33.1 (7.1)	40.4 (6.8)	.010*
Male, n (%)	7 (100%)	35 (58.3%)	.041*
Right‐sided pathology, n (%)	1 (14.3%)	38 (63.3%)	.013*
Dominant arm, n (%)	2 (28.6%)	40 (66.7%)	.097
Traumatic mechanism, n (%)	1 (14.3%)	24 (40.0%)	.245
Combat arms, n (%)	6 (85.7%)	35 (58.3%)	.239
Time to surgery, months	35.6 (38.7)	30.3 (54.9)	.803
Mean tear size, mm	1.6 (0.79)	1.6 (0.61)	.888
Follow‐up, months	74.7 (32.5)	70.6 (25.4)	.699
Concomitant procedures			
Subacromial decompression, n (%)	7 (100%)	60 (100%)	‐
Biceps tenotomy/Tenodesis, n (%)	7 (100%)	53 (88.3%)	.336
Acromioclavicular joint resection, n (%)	1 (14.3%)	24 (40.0%)	.245
Rotator interval release, n (%)	1 (14.3%)	14 (23.3%)	1.00
Patient‐reported outcome measures			
VAS			
Preoperative	7.9 (1.8)	8.2 (1.3)	.591
Postoperative	5.3 (3.1)	1.3 (2.1)	.001*
SANE			
Preoperative	52.1 (16.8)	48.6 (19.6)	.640
Postoperative	64.3 (17.9)	87.0 (14.7)	.003*
ASES			
Preoperative	48.0 (11.0)	47.4 (10.1)	.888
Postoperative	59.8 (13.0)	90.7 (11.4)	.001*

*Note*: Scores in patients with tear progression were collected prior to rotator cuff repair.

ASES, American Shoulder and Elbow Surgeons score; SANE, Single Assessment Numeric Evaluation; VAS, Visual Analog Scale.

* Indicates statistical significance.

## DISCUSSION

In the present study, debridement of articular‐sided Ellman grade I supraspinatus PT‐RCT in conjunction with associated procedures among active‐duty military personnel resulted in significant improvements in pain scores and PROMs as well as high rates of return to unrestricted active duty and sport, despite high rates of atraumatic progression to full‐thickness tears observed at 10.4%. Additionally, the cohort that experienced progression tended to be younger and more male predominate on average. The findings from the present study indicate that debridement of Ellman grade I PT‐RCT among ADSM offers benefits and relatively high rates of return to duty and sport; however, informing patients of the risk of tear progression is important to preoperative decision‐making and counseling as well as postoperative surveillance.

Arthroscopic debridement of both articular‐ and bursal‐sided PT‐RCT has become a widely accepted treatment option yielding favorable outcomes in pain reduction and functional restoration.^4^, ^5^, ^8^, ^9^, ^10^, ^11^, ^12^, ^16^ In their comparison of debridement versus repair among patients with articular‐sided Ellman grade II and III PT‐RCT, Brockmeyer et al. report higher PROMs among patients undergoing debridement, although rates of patient satisfaction and acceptable shoulder function remained high among both groups.^8^ Evidence exists that the benefits associated with debridement alone may be time dependent. In their study of patients with Ellman grade II bursal PT‐RCT, Zhang et al. found significantly greater improvements in VAS, ASES, Constant‐Murley, and UCLA scores among patients who underwent debridement at 6 months but not at other follow‐up intervals.^12^ Although there is ample evidence to suggest that low‐grade PT‐RCT can be successfully treated with debridement, the population in the present study is approximately a decade younger than many in existing research.^8^, ^12^ Regardless of age, the significant improvements in postoperative PROMs without observed differences in postoperative ROM in the present study involving patients with a mean age of 40 suggests that tear debridement may be beneficial in maintaining current shoulder mobility or preventing further deterioration of shoulder mobility. Subsequently, in the context of improvements in pain, functional scores, and preserved postoperative ROM, debridement of low‐grade PT‐RCT appears to offer a viable treatment option to improve shoulder pain and function, even among a highly active population.

Beyond improvements in pain and functional outcomes, understanding rates of return to active duty and physical activity is important to preoperative counseling among a young, active population. Existing literature regarding outcomes among athletes undergoing debridement of PT‐RCTs describes benefits in improving symptoms with varying abilities to return to play.^17^ In their case series of professional baseball pitchers who underwent debridement of PT‐RCT for tears of any depth (74% of which were classified as Ellman grade I), Reynolds et al. report a 76% return‐to‐sport rate, with 21% returning at a lower level of play, 54% returning at the same level of play, and 1% returning at a higher level of play.^11^ Similarly, in their analysis of a variety of athletes younger than 40 years who underwent debridement for PT‐RCTs, 91% of which were articular‐sided, Payne et al. report good to excellent results among 86% of patients with acute injuries and 66% of patients with chronic injuries. The overall return‐to‐sport rate was 51%, with 64% of patients with acute injuries able to return to sport versus 45% of patients with chronic injuries.^18^ In the present study, rates of return to unrestricted duty and sport were slightly higher, at 88% and 85% respectively). Additionally, the high rates of combat arms military occupational specialties such as infantry, which are typically associated with higher physical demands than noncombat arm occupations, is similarly reassuring that patients are able to reach high levels of function. Although return to unrestricted active duty and recreational sports is not directly comparable to return to preinjury levels of sport in athletes, the high level of return to duty observed in this study is promising when considering the physical demands of active‐duty military personnel, who sustain high occupational shoulder demand.^15^, ^19^, ^20^, ^21^, ^22^, ^23^, ^24^, ^25^ Unlike athletes, whose ability to return to sport at the same or higher level may be threatened by debridement of low‐grade PT‐RCT tears, high rates of return to unrestricted active duty suggests that arthroscopic debridement of these lesions enables ADSM to achieve adequate occupational function postoperatively.

Subacromial impingement is a common concomitant finding alongside PT‐RCT, and subacromial decompression is commonly performed alongside rotator cuff debridement.^8^, ^12^, ^18^, ^26^, ^27^, ^28^, ^29^ All patients in the present study were found to have subacromial inflammation and underwent concomitant ASAD. Additionally, more than half (56.7%) showed long head of the biceps tendon pathology and underwent concomitant biceps tenodesis or tenotomy. At nearly 20 years of follow‐up, Jaeger et al. report favorable outcomes with subacromial decompression and debridement of PT‐RCT, and, among their cohort of patients with Ellman grade I tears, Liem et al. report good to excellent results with acromioplasty alone.^27^, ^28^ In the present patient population, the combination of ASAD with rotator cuff debridement and, frequently, biceps tenodesis or tenotomy appears to offer symptom relief and functional improvements sufficient to enable return to active duty. Further research will help to clarify the degree to which concomitant procedures, especially ASAD, improves outcomes after arthroscopic debridement of low‐grade PT‐RCT involving the supraspinatus. Additionally, future research will help better elucidate the role of adjunctive procedures versus rotator cuff debridement in isolation to facilitate clinical improvement among this patient population.

Despite significant improvements in pain and functional outcomes, it is important to note that the progression of partial‐thickness tears to symptomatic full‐thickness tears remains a concern. In the present study, 10.4% of patients experienced progression to a symptomatic full‐thickness rotator cuff tear over the follow‐up period and were indicated for arthroscopic rotator cuff repair. Despite the small population size in the present study, this rate of progression roughly falls within the wide and highly variable range of reported rates in existing literature, with a systematic review by Strauss et al.^13^ reporting progression rates ranging from 6.5 to 34.6% after debridement of PT‐RCT less than 50% of the tendon thickness. Additionally, existing evidence suggests that high physical work level is associated with tear progression.^30^ Although the proportion of patients with combat arms occupations was statistically similar between cohorts, patients who experienced tear progression versus their stable counterparts showed a younger average age (33 vs 40 years), a greater proportion of male individuals (100% vs 58%), and a greater proportion of left‐sided tears (86% vs 37%). Given the potential for an increased risk for tear progression among a younger and more commonly male cohort, preoperative counseling should include a discussion of the risk for tear progression. Ultimately, the results from the present study suggest that patients who are indicated for arthroscopic rotator cuff debridement require careful patient selection, shared decision‐making, and close postoperative monitoring to optimize long‐term outcomes and minimize time spent with duty restrictions in this population.

### Limitations

This study is not without limitations. The retrospective design with the lack of an associated control group introduces the potential for selection bias and limits the ability to establish causal relationships. Additionally, all patients who continued to endorse symptoms after 3 months of nonoperative treatment were offered surgical intervention as standard for the surgeon's practice; however, this practice may introduce selection bias in the patients who elect to proceed with surgery versus continue with nonoperative measures. The study relied on medical records and patient‐reported outcomes, which are subject to documentation inconsistencies and recall bias, as well as ROM measurement, which is inherently subject to possible measurement error. Variability in follow‐up duration among patients (ranging from 24 to 120 months) may have influenced the uniformity of the data collected. This study also exclusively included predominately male active‐duty military personnel younger than 50 years without preexisting instability, subscapularis tears, or glenohumeral osteochondral defects, limiting generalizability to broader patient populations. Furthermore, all patients underwent ASAD and many underwent biceps tenotomy/tenodesis and ACJR, which may confound the true efficacy of the rotator cuff debridement in yielding clinical improvement. Additionally, asymptomatic progression to full‐thickness rotator cuff tears or patients who did not present for further evaluation were not captured within this study. The comparative analysis between patients who experienced symptomatic tear progression and those who did not similarly introduces selection bias in assessing these results.

## CONCLUSIONS

Arthroscopic debridement of Ellman grade I, articular‐sided supraspinatus PT‐RCT with adjunctive procedures in ADSM younger than 50 years shows substantial pain relief, functional recovery, and return to military service and sport. Despite improvements in pain relief and functional outcomes, approximately 1 in 10 patients experienced atraumatic tear progression to a symptomatic full‐thickness rotator cuff tear and ultimately underwent rotator cuff repair.

## DISCLOSURES

The authors (R.G., S.H., I.S.R., J.P.S.) declare the following financial interests/personal relationships which may be considered potential competing interests: R.G. reports a relationship with Enovis Corporation that includes: speaking and lecture fees; reports a relationship with Smith & Nephew that includes: consulting or advisory. Given his role as an editorial board member for *Arthroscopy*, he had no involvement in the peer review of this article and had no access to information regarding its peer review. Full responsibility for the editorial process for this article was delegated to another journal editor. S.H. reports a relationship with Pinnacle, that includes: travel reimbursement. N.P. reports a relationship with MiTek that includes: consulting or advisory. I.S.R. reports a relationship with Smith & Nephew that includes: travel reimbursement; reports a relationship with Legacy Ortho LLC that includes: travel reimbursement. J.P.S. serves as an editorial board member for *Arthroscopy*. The other authors (B.S., A.B.S., R.C., J.T.) declare that they have no known competing financial interests or personal relationships that could have appeared to influence the work reported in this article.
